# Screening and verifying the mutations in the *LDLR* and *APOB* genes in a Chinese family with familial hypercholesterolemia

**DOI:** 10.1186/s12944-023-01935-8

**Published:** 2023-10-18

**Authors:** Xian Lv, Chunyue Wang, Lu Liu, Guoqing Yin, Wen Zhang, Fuad A. Abdu, Tingting Shi, Qingfeng Zhang, Wenliang Che

**Affiliations:** 1 Department of Cardiology, Shanghai Tenth People’s Hospital, Tongji University School of Medicine, 301 Yanchang Road, Shanghai, 200072 China; 2 Department of Cardiology, Shanghai Tenth People’s Hospital Chongming Branch, Tongji University School of Medicine, Shanghai, China; 3 Key Laboratory of Spine and Spinal Cord Injury Repair and Regeneration of Ministry of Education, Tongji Hospital, Clinical Center for Brain and Spinal Cord Research, School of Medicine, Tongji University, 1239 Siping Road, Shanghai, 200092 China

**Keywords:** Familial hypercholesterolemia, *LDLR*, *APOB*, Pathogenic mutation, PCSK9 inhibitor

## Abstract

**Background:**

Familial hypercholesterolemia (FH) is an autosomal dominant genetic disorder. The primary objective of this study was to identify the major pathogenic mutations in a Chinese family with FH.

**Methods:**

Whole-genome sequencing (WGS) was used to identify variants of FH-related genes, including low-density lipoprotein receptor *(LDLR)*, apolipoprotein B *(APOB)*, and proprotein convertase subtilisin/kexin 9 *(PCSK9)*. Bioinformatics software was used to predict signal peptides, transmembrane structures, and spatial construction information of the mutated sequences. Western blotting was performed on the mutant protein to determine the presence of the major structural domains of the LDLR. The *PCSK9* and *APOB* genes were screened and analyzed. Moreover, the proband and his brother were treated with a PCSK9 inhibitor for 1 year, and the effect of the treatment on lipid levels was assessed.

**Results:**

WGS revealed two potentially pathogenic mutations in the *LDLR* gene. One was a novel mutation, c.497delinsGGATCCCCCAGCTGCATCCCCCAG (p. Ala166fs), and the other was a known pathogenic mutation, c.2054C>T (p. Pro685Leu). Bioinformatics prediction and in vitro experiments revealed that the novel mutation could not be expressed on the cell membrane. Numerous gene variants were identified in the *APOB* gene that may have a significant impact on the family members with FH. Thus, it is suggested that the severe manifestation of FH in the proband primarily resulted from the cumulative genetic effects of variants in both *LDLR* and *APOB*. However, a subsequent study indicated that treatment with a PCSK9 inhibitor (Evolocumab) did not significantly reduce the blood lipid levels in the proband or his brother.

**Conclusions:**

The cumulative effect of *LDLR* and *APOB* variants was the primary cause of elevated blood lipid levels in this family. However, PCSK9 inhibitor therapy did not appear to be beneficial for the proband. This study emphasizes the importance of genetic testing in determining the most suitable treatment options for patients with FH.

**Supplementary Information:**

The online version contains supplementary material available at 10.1186/s12944-023-01935-8.

## Background

Familial hypercholesterolemia (FH) is a prevalent autosomal genetic disorder associated with gene-dosage effects [1]. The most common cause of FH is the presence of pathogenic gene mutations in the low-density lipoprotein receptor (*LDLR*), apolipoprotein B (*APOB*), and proprotein convertase subtilisin/kexin 9 (*PCSK9*). Genetic diagnosis is currently the gold standard for the diagnosis of FH [2]. However, previous studies have shown that in many countries, less than 1% of patients with FH receive an accurate and official diagnosis [3].

Among the pathogenic gene mutations in FH, *LDLR* gene mutations account for 86–88%, *APOB* gene mutations account for 12%, and *PCSK9* gene mutations account for less than 1% [4]. As FH involves the accumulation of genetic effects, patients may have mutations in more than one gene associated with FH. LDLR is a liver cell surface membrane protein that is mainly responsible for transporting low-density lipoprotein (LDL) to lysosomes for metabolism. Subsequently, the transferred LDLR resumes its function on the surface of liver cells [5]. LDLR primarily consists of six structural domains:1) a signal peptide located at the N-terminus, which guides the protein to the endoplasmic reticulum; 2) a ligand-binding domain, which is responsible for binding to LDL and is typically composed of multiple repeated structural units; and 3) epidermal growth factor (EGF)-like repeats containing multiple repeated EGF-like structural units, such as EGF-A and EGF-B. These domains participate in regulating the binding of LDL and its uptake by cells; 4) the O-linked sugar domain, which plays a significant role in the function and stability of LDLR; 5) the transmembrane domain located in the transmembrane region of LDLR, which anchors the protein within the cell membrane; and 6) the cytoplasmic domain situated at the C-terminus, which functions within the cell and participates in LDLR internalization and signal transduction [6]. Pathogenic mutations in *APOB* are less severe than *LDLR* mutations and mostly occur in the LDLR-binding domain [7]. Each LDL particle must bind to the apoB-100 protein, and LDLR binds to the ligand-binding domain of apoB-100, facilitating the internalization of LDL particles [6].

The severity of clinical symptoms in FH patients depends on the plasma low-density lipoprotein cholesterol (LDL-C) levels. The prolonged elevation of blood lipid levels can lead to the development of xanthomas, corneal arcus, and premature coronary heart disease [8]. The main risk factor of FH is atherosclerotic cardiovascular disease [9]. Therefore, it is imperative to reduce LDL-C levels in patients with FH. Based on genotype and average blood lipid levels, the order of severity is as follows: heterozygous FH < double heterozygote (*LDLR* + *APOB* mutation or *PCSK9* gain-of-function mutation) < homozygous *APOB* or *PCSK9* gain-of-function mutation < homozygous low-density lipoprotein receptor adaptor protein 1 (*LDLRAP1*) or *LDLR*-defective mutations < compound heterozygote *LDLR*-defective + *LDLR*-negative mutations < homozygous *LDLR*-negative mutations [10]. Hence, the primary therapy for FH is to reduce blood lipid levels. A wide range of lipid-lowering treatment options are available for FH, including medication (e.g., statins, ezetimibe, and PCSK9 inhibitors), lipoprotein apheresis, and surgical interventions (e.g., liver transplantation and other surgical procedures) [10]. PCSK9 inhibitors are highly effective in patients with FH [11]. However, some patients remain resistant to the current lipid-lowering therapies, highlighting the need for novel therapies.

In the present study, a novel *LDLR* mutation (c.497delinsGGATCCCCCAGCTGCATCCCCCAG: p. Ala166fs) was identified. This study, for the first time, confirmed through in vitro experiments and bioinformatics predictions that the mutation could not be properly expressed on the cell membrane. Additionally, analysis of the *APOB* and *PCSK9* genes suggested that variants in the *APOB* gene played an important role in this family. The suboptimal therapeutic efficacy of a PCSK9 inhibitor (Evolocumab) provides valuable insights into clinical treatments and emphasizes the potential of genetic testing to guide medication selection.

## Materials and methods

### Study population, inclusion criteria, and sample collection

Written informed consent was obtained from all patients. The study protocol was in accordance with the ethical guidelines of the 1975 Declaration of Helsinki. The Institutional Ethics Committee of the Shanghai Tenth People’s Hospital approved the human research procedures (22K127).

Dutch Lipid Clinic Network (DLCN) diagnostic criteria were used in this study [12]. Whole blood samples from patients’ families were collected in anticoagulant tubes and stored in freezers.

### Whole-genome sequencing (WGS)

Genomic DNA was extracted from the blood samples using an Omega Blood DNA Kit (Omega, USA). WGS was performed and analyzed by Novogene Bioinformatics Technology Co., Ltd. Sequencing was performed using the PE150 high-throughput sequencing method on the Illumina platform. Following sequencing, the raw sequences were subjected to information analysis to assess data quality and determine whether they met the standards. Valid sequencing data were aligned to the reference genome (GRCh37/hg19/GRCh38) and sorted based on the alignment results. Duplicate reads were marked using Sambamba software. Finally, statistics on the coverage, depth, and other parameters were calculated using the alignment results with marked duplicates. Typically, human sample sequencing achieves a mapping rate of over 95%. When a position reached a read depth of 10× or higher, the single-nucleotide polymorphisms detected at that location were considered reliable. ANNOVAR was used to annotate variant call formats obtained from previous studies. It uses the latest information to annotate gene variations detected across multiple genomes, thereby providing functional annotations.

### RNA extraction and plasmid construction

Total RNA was extracted from TRIzol-preserved samples from family members. Reverse transcription was performed using reverse transcription kit (R312-01, Vazyme, Nanjing) to obtain the cDNA fragments. The amplification was performed using a high-fidelity polymerase chain reaction (PCR) enzyme (P520-01, Vazyme, Nanjing). The following PCR primers were used to amplify the novel mutation sequence in the coding sequence (CDS) of *LDLR*: FP, ATGGGGCCCTGGGGCTGG; and RP, TCATCCGAGCCATCTTCG. Total amount of template cDNA was the same for both the normal and proband samples. The amplification results were analyzed by gel electrophoresis and Sanger sequencing.

The mutant sequence of the proband was obtained using reverse transcription-polymerase chain reaction (RT-PCR). The amplified fragment was inserted into pcDNA3.1, using homologous recombination. Additionally, we fused an HA tag sequence to the C-terminus of both the mutant and wild-type (WT) plasmids.

### Sanger sequencing

To verify the variants of the exons in the *LDLR* gene, PCR amplification of cDNA was performed. PCR primers were designed based on the exon from which the mutation was identified. Each family member was systematically screened for variants using Sanger sequencing. Chromas software was used to analyze the sequencing data.

### Cell culture and plasmid transfection

HEK293 cells were cultured in DMEM medium supplemented with 10% fetal bovine serum, 100 units/mL penicillin, and 100 µg/mL streptomycin in a 37 °C incubator containing 5% CO_2_. HEK293 cells were grown in six-well culture plates for plasmid transfection, and the cell density was adjusted to 60–70%. Three groups were used: negative control (NC) group, in which empty plasmids were transfected into HEK293 cells; WT group, in which HEK293 cells were transfected with WT plasmids; and mutant group, in which mutant plasmids were transfected into HEK293 cells.

Plasmid DNA was mixed with Lipofectamine 3000 (L3000075, Thermo Fisher Scientific) according to the manufacturer’s instructions and incubated for 10–15 min at room temperature. To maximize LDLR expression, the mixture was incubated for 48–72 h.

### Validate the expression of LDLR

HEK293 cells were lysed in RIPA buffer containing protease inhibitors after plasmid transfection. The protein concentration in each sample was measured using the BCA protein assay kit. The samples were boiled in SDS-PAGE loading buffer at 100 °C for 5–10 min and separated using SDS-PAGE. The separated proteins were transferred to PVDF membranes and blocked in a solution of 5% milk in PBST for 1 h at room temperature. The membrane was incubated with the primary antibodies overnight at 4℃. The primary antibodies used are as follows: GAPDH (1:6000, 60004-1-Ig, Proteintech), LDLR (1:1000, ab286156, Abcam), HA (1:3000, 51064-2-AP, Proteintech). The membranes were incubated with the corresponding rabbit or mouse secondary antibodies at room temperature for one hour. Finally, Amersham Imager 600 software was used to measure the sample signals.

### Bioinformatics methods for protein structure prediction

SignalP predicts whether a protein sequence contains a signal peptide based on amino acid composition, hydrophobicity, and position [13]. This information helps determine whether the protein can proceed to the translation stage. TMHMM is a tool based on Hidden Markov Models for predicting the transmembrane regions of proteins. For unknown protein sequences, TMHMM uses information such as amino acid composition, hydrophobicity, and isoelectric point to determine whether the protein sequence contains transmembrane regions [14]. SWISS-MODEL is a tool based on comparative modeling principles to predict three-dimensional protein structures. It generates predicted protein structures by comparing the target protein sequence to known structures and template proteins [15].

## Results

### Clinical information and pedigree investigation of the proband

**Fig. 1:**
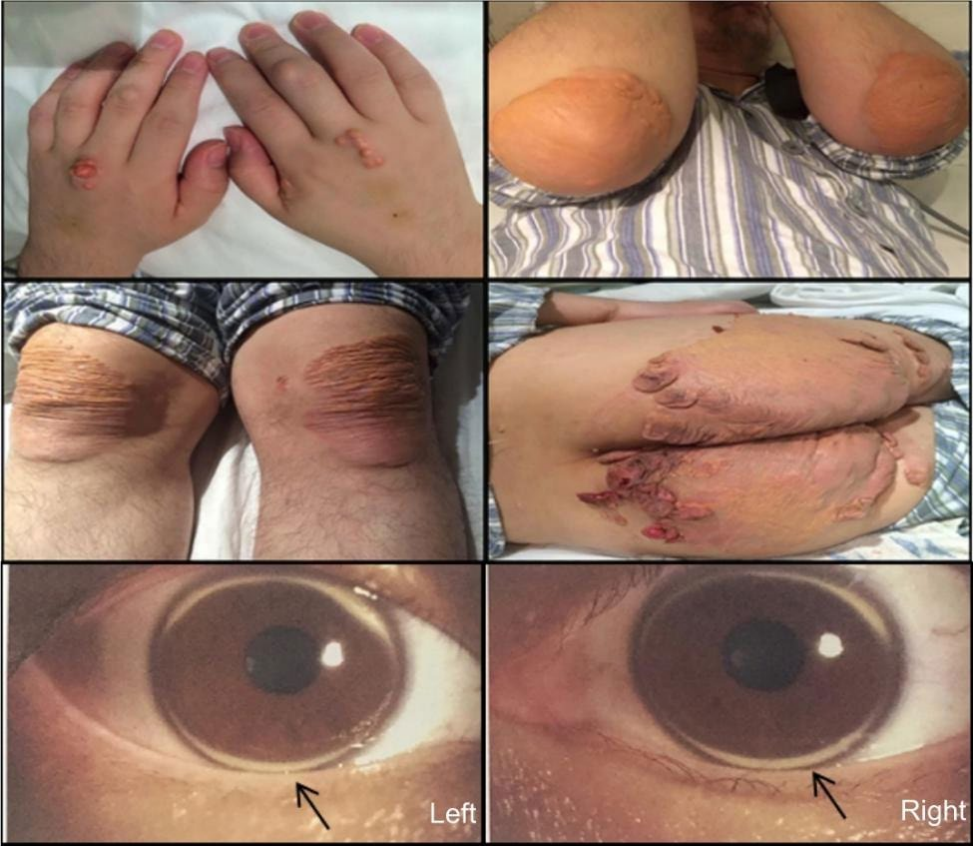
The clinical information of the proband. The proband exhibited xanthomas on the elbows (4 × 5 cm), knees (6 × 7 cm), hips (10 × 10 cm), and corneal arch in both eyes

The proband was diagnosed with severe hyperlipidemia and had typical clinical symptoms of FH, including corneal arcs and xanthoma on the skin of the elbow (4 × 5 cm), hip (10 × 10 cm), and tendon (6 × 7 cm) (Fig. 1). Dyslipidemia in the proband’s family was primarily characterized by elevated LDL-C levels. Unfortunately, the proband’s grandmother (I2) died at the age of 77 because of myocardial infarction. However, the proband’s grandfather (I3), maternal grandfather (I1) and uncle (II2) had normal blood lipid levels. The clinical biochemical data of the family members are shown in Table 1.

**Table 1:** Clinical biochemical data of family members with FH

Families	Age (years)	TC (mmol/L)	HDL-C (mmol/L)	LDL-C (mmol/L)	TG (mmol/L)	DLCN scores
Proband	31	16.84	0.87	13.44	5.79	29
Father	62	7.31	1.23	5.67	0.72	11
Mother	62	10.64	2.09	7.80	0.68	16
Brother	22	18.04	0.64	15.00	0.96	27
Aunt	63	9.08	1.47	7.58	0.86	13

*Abbreviations*: *TC*, total cholesterol; *HDL-C*, high-density lipoprotein cholesterol; *LDL-C*, low-density lipoprotein cholesterol; *TG*, triglycerides; *DLCN*, Dutch Lipid Clinic Network

### Genetic mutation analysis

**Fig. 2:**
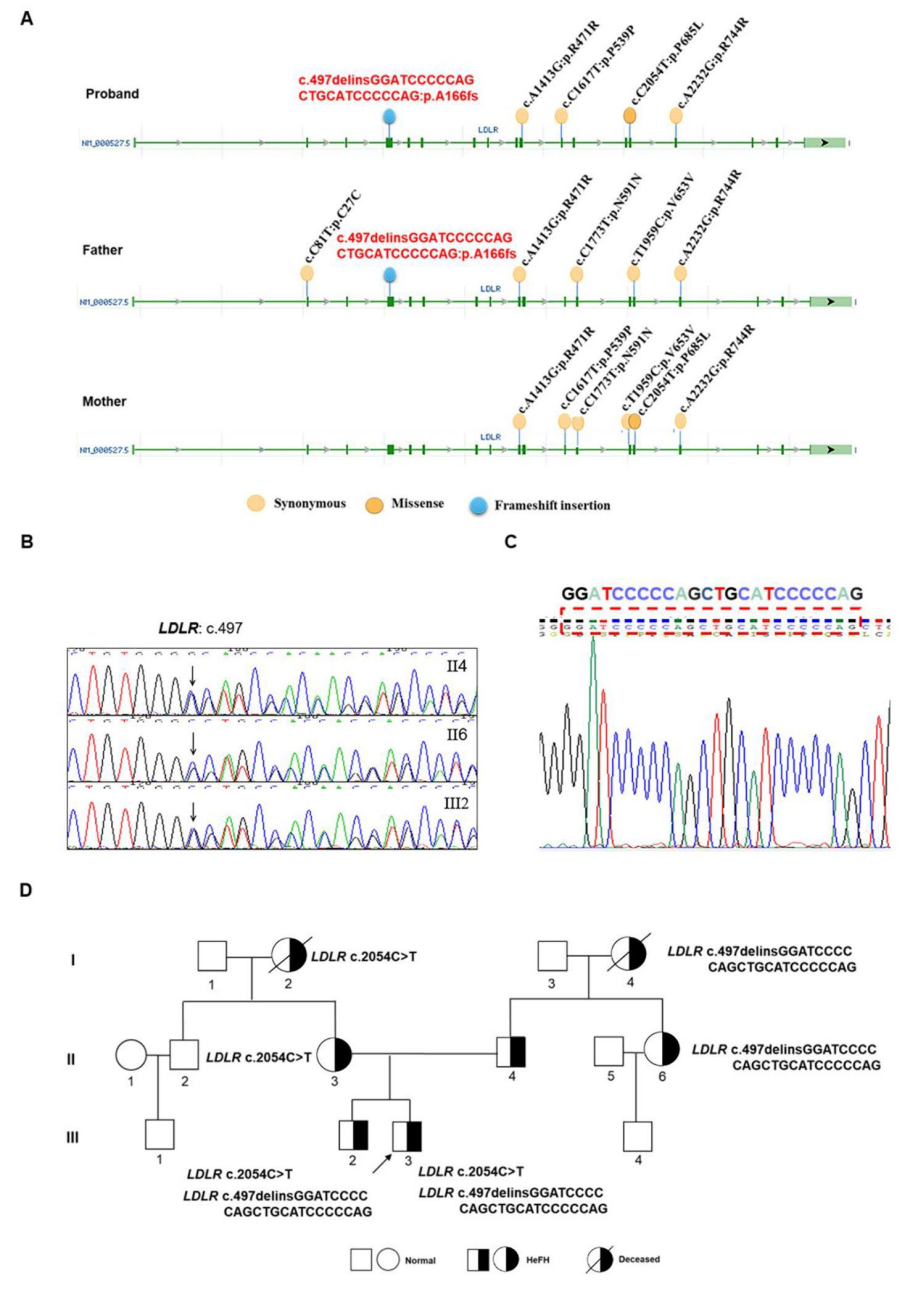
Sequencing results and family tree. (**A**) *LDLR* variants were identified in this family through WGS. (**B**) Sanger sequencing results of the proband’s father (II4), aunt (II6), and younger brother (III2) were obtained. (**C**) The sequencing results of the proband from the TA-clone of *LDLR* revealed a novel frameshift insertion. (**D**) The mutations of *LDLR* were displayed on the family tree

WGS was performed on three individuals in this family. From the results of WGS, we performed screening for FH-related genes (*LDLR*, *APOB*, *PCSK9*). The *LDLR* gene has been found to have various variants including synonymous, missense, and frameshift insertion. The results for the *LDLR* variants in the proband and his parents are illustrated in Fig. 2A. The functional prediction results of the *LDLR* variants found in the proband were analyzed using the database of *LDLR* mutations (http://www.lovd.nl/LDLR) and are presented in Table 2. According to the database, the *LDLR* c.2054C>T (p. Pro685Leu) mutation was a pathogenic mutation inherited from the proband’s mother.

**Table 2:** Prediction of the function of *LDLR* gene variants in the proband

Numbers	DNA change	Protein	P-domain	Clinical classification	Affects function
1	c.1413A>G	p. R471R	LDLR class B2	Benign	Does not affect function
2	c.1617C>T	p. P539P	LDLR class B4	Benign	Does not affect function
3	c.2054C>T	p. P685L	EGF-like 3	Likely pathogenic	Probably affects function
4	c.2232A>G	p. R744R	-	-	Unreported

Sanger sequencing was used to confirm the mutation sites in the *LDLR* gene. The results showed similar chaotic doublets in exon 4 of the *LDLR* gene in the proband’s father (II4), aunt (II6), and younger brother (III2) (Fig. 2B). TA-cloning was performed to verify the mutation and it was discovered that the mutation in exon 4 of *LDLR* (c.497delinsGGATCCCCCAGCTGCATCCCCCAG: p. Ala166fs) in proband had never been previously reported (Fig. 2C). The Sanger sequencing results are consistent with the WGS results. Sanger sequencing was also conducted to validate the presence of the mutation in other family members, and it was found that the mutation was also present in father (II4), aunt (II6), and younger brother (III2). The pedigree map of the mutations in *LDLR* is shown in Fig. 2D. Based on the above results, we believe that the pathogenic mutation c.2054C>T (p. Pro685Leu) and the newly discovered mutation c.497delinsGGATCCCCCAGCTGCATCCCCCAG (p. Ala166fs) may be the causative mutations of FH in this family.

### Structural prediction and expression of the mutant LDLR protein

**Fig. 3:**
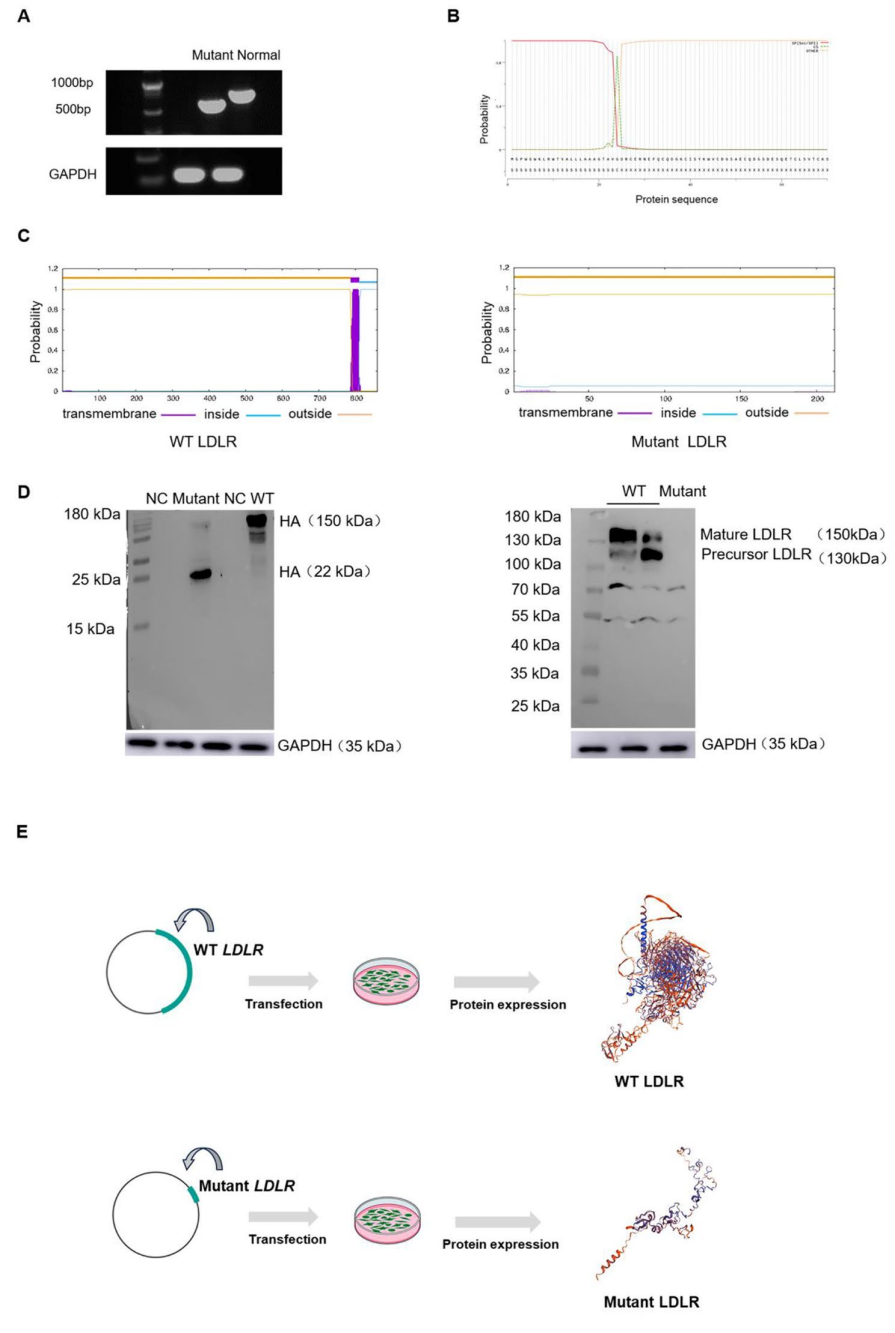
Bioinformatics prediction and in vitro validation of the mutated sequence of LDLR. (**A**) The gel electrophoresis image showed the full-length CDS region (558 bp) of the mutation fragment and the CDS region of the normal *LDLR* (exon 1–4). (**B**) SignalP was used to predict the presence of a complete signal peptide in the mutated LDLR. (**C**) TMHMM confirmed that the mutant sequence does not contain any transmembrane domains. (**D**) Western blotting was used to detect the mutant LDLR protein, which had a size of 22 kDa. However, it did not bind to the anti-LDLR antibody. (**E**) SWISS-MODEL was used to generate spatial structures of both the normal LDLR and the mutated LDLR protein

In contrast to previously reported single-nucleotide mutations, this frameshift insertion led to the premature appearance of a stop codon. To investigate whether the mutation could progress to the translation stage, RT-PCR was performed using equal amounts of the normal template and the proband’s sample. The resulting agarose gel electrophoresis image indicated that mRNA containing this mutation was not degraded (Fig. 3A). Considering the intactness of the N-terminal sequence of the mutated fragment, SignalP predicted that this fragment contained a signal peptide with a translation initiation signal (Fig. 3B).

The transmembrane region of the mutated protein was predicted using TMHMM, which revealed an absence of transmembrane domains, suggesting that this protein may not form a transmembrane protein (Fig. 3C). Based on the bioinformatics analysis, it was hypothesized that the mutated sequence may progress to the protein translation stage, but fail to generate a mature LDLR protein. Because protein quality control occurs in the endoplasmic reticulum, expression of the mutated protein requires a stable conformation and functionality. To confirm the predominant structure of the mutant protein, LDLR antibodies capable of recognizing LDLR-like sequences such as EGF-like domains, LDLR class A domains, or LDLR class B repeats were used. These sequences contribute to the structural characteristics and function of LDLR. Western blotting was used to demonstrate that the HEK293 cells used in this study do not express the LDLR protein (Supplementary Fig. 1). The CDS fragment of the mutant sequence derived from the proband’s cDNA was cloned into a pcDNA3.1 vector and protein expression was confirmed by detecting the tagged HA protein. However, the same anti-LDLR antibody failed to detect it (Fig. 3D). This result indicates that the mutant protein lacks the major functional domains of LDLR.

The SWISS-MODEL website, which uses the theory of protein homology modeling, is used to predict protein structures. Based on this prediction, the mutant protein was substantially shorter and simpler than normal LDLR (Fig. 3E). Based on the bioinformatics predictions and in vitro experiments, it was observed that this novel mutation altered the primary structure of the LDLR protein, resulting in an inability to form a protein with functional LDLR domains. In additionally, the spatial structure of the proteins was altered. Mutation c.2054C>T (p. Pro685Leu) is located in the EGF-like domain of LDLR and is involved in LDL uptake. Overall results, both mutations in the *LDLR* gene were pathogenic.

### Screening of *APOB* and *PCSK9* genes

**Fig. 4:**
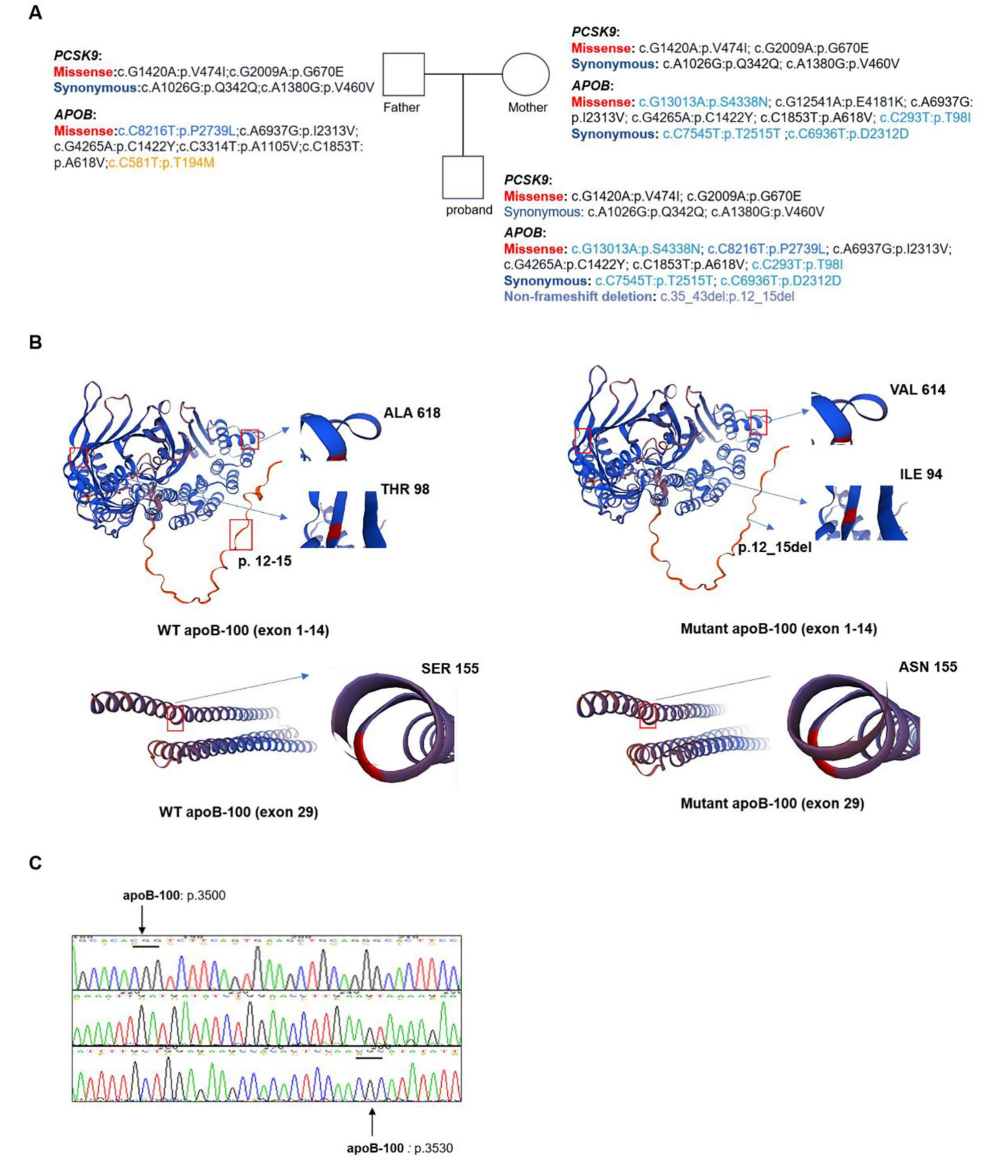
Summary of gene variants in *APOB* and *PCSK9.* (**A**) Based on the results of WGS, the mutations in the *APOB* and *PCSK9* genes for the proband and the parents were compiled. (**B**) SWISS-MODEL was employed to generate a structure depicting the variants located in *APOB* (Exon 1, exon 4, exon 14 and exon 29). Variants in exon1 and exon 29 led to a change in the spatial conformation of the protein. (**C**) Sanger sequencing confirmed that the proband had no nucleotide variants at the pathogenic mutation sites prone to occur in the apoB-100 protein (p.3500–3530)

To identify pathogenic variants in this family, we investigated and analyzed variants in the *PCSK9* and *APOB* genes (Fig. 4A). Variants in the *PCSK9* gene located outside the major functional domains are unlikely to be the primary underlying cause of its pathogenesis. Based on the American College of Medical Genetics and Genomics (ACMG) classification, most of the variants identified in the *APOB* gene are categorized as either benign or likely benign. This classification indicates that these variants are not likely to cause disease based on the available evidence. Uncertain significance means that it is a category of mutations that have the potential to be disease-causing. Additionally, there may be some unreported variations in *APOB* gene. Exons 26 and 29 of the *APOB* gene primarily encode the structural domains involved in the clearance of LDL particles. The proband had five variants (c.6936C>T: p. Asp2312Asp; c.7545C>T: p. Thr2515Thr; c.4265G>A: p. Cys1422 Tyr; c.6937A>G: p. Ile2313Val; c.8216C>T: p. Pro2739Leu) in exon 26 of the *APOB* gene. Among these variants, the mutation at position 6937 is homozygous. According to the ACMG criteria, this mutation (c.6937A>G: p. Ile2313Val) is classified as a variant of benign significance. Due to the large size and complex nature of apoB-100 protein, SWISS-MODEL is unable to generate a complete structure, especially for the region encoded by exon 26. Spatial structure predictions for variants located in exon 1 (c.35_43del: p.12_15del), exon 4 (c.293C>T: p. Thr98Ile), exon 14 (c.1853C>T: p. Ala618Val), and exon 29 (c.13013G>A: p. Ser4338Asn) of the *APOB* gene revealed that the variants occurring in exon 1 and exon 29 alter the protein’s spatial structure (Fig. 4B).

In previous studies, the region encompassing amino acid positions 3500–3530 in the apoB-100 protein was found to be prone to pathogenic mutations. Sanger sequencing confirmed that no variants were present at this locus in the proband (Fig. 4C). The table lists all variants and information on the FH-related genes (*LDLR*, *APOB*, and *PCSK9*) identified in the pedigree (Table 3). The clinical classification information of the variants is derived from using the ClinVar database. Assessing the population frequency of the variants observed in this study by utilizing the gnomAD website.

**Table 3:** Information on FH-related gene (*LDLR*, *APOB*, and *PCSK9*) variants in the family

Numbers	Gene Name	ID	DNA change	Mutation type	Clinical classification	Allele Frequency
1	*LDLR*	rs28942084	c.2054C>T	Missense	Likely Pathogenic	0.00003
2	*LDLR*	rs2228671	c.81C>T	Synonymous	Benign	-
3	*LDLR*	rs5930	c.1413A>G	Synonymous	Benign	0.6336
4	*LDLR*	rs5929	c.1617C>T	Synonymous	Benign	0.07550
5	*LDLR*	rs688	c.1773C>T	Synonymous	Benign	0.3825
6	*LDLR*	rs5925	c.1959T>C	Synonymous	Benign	0.4150
7	*LDLR*	rs5927	c.2232A>G	Synonymous	Unclassified	0.7776
8	*LDLR*	-	c.497delinsGGATCCCCCAGCTGCATCCCCCAG	Frameshift insertion	Unreported	-
9	*APOB*	rs17240441	c.35_43del	Non-frameshift deletion	Unreported	-
10	*APOB*	rs1367117	c.293C>T	Missense	Benign/Likely benign	0.2562
11	*APOB*	rs13306198	c.581C>T	Missense	Benign/Likely benign	0.00470
12	*APOB*	rs679899	c.1853C>T	Missense	Benign/Likely benign	0.4818
13	*APOB*	rs185540148	c.3314C>T	Missense	Unreported	0.000007075
14	*APOB*	rs568413	c.4265G > A	Missense	Unreported	0.9998
15	*APOB*	rs584542	c.6937A>G	Missense	Benign	-
16	*APOB*	rs676210	c.8216C>T	Missense	Unreported	0.2866
17	*APOB*	rs1042031	c.12541G>A	Missense	Uncertain significance	-
18	*APOB*	rs1042034	c.13013G>A	Missense	Benign	0.7119
19	*APOB*	rs693	c.7545C>T	Synonymous	Benign	0.3877
20	*APOB*	rs1041968	c.6936C> T	Synonymous	Benign	0.3872
21	*PCSK9*	rs562556	c.1420G>A	Missense	Unreported	0.8539
22	*PCSK9*	rs505151	c.2009G>A	Missense	Unreported	0.9417
23	*PCSK9*	rs509504	c.1026A>G	Synonymous	Benign	0.9942
24	*PCSK9*	rs540796	c.1380A>G	Synonymous	Benign	0.8539

*Abbreviations*: *APOB*, apolipoprotein B; *LDLR*, low-density lipoprotein receptor; *PCSK9*, proprotein convertase subtilisin/kexin 9

### The therapeutic effects of the PCSK9 inhibitor

**Fig. 5:**
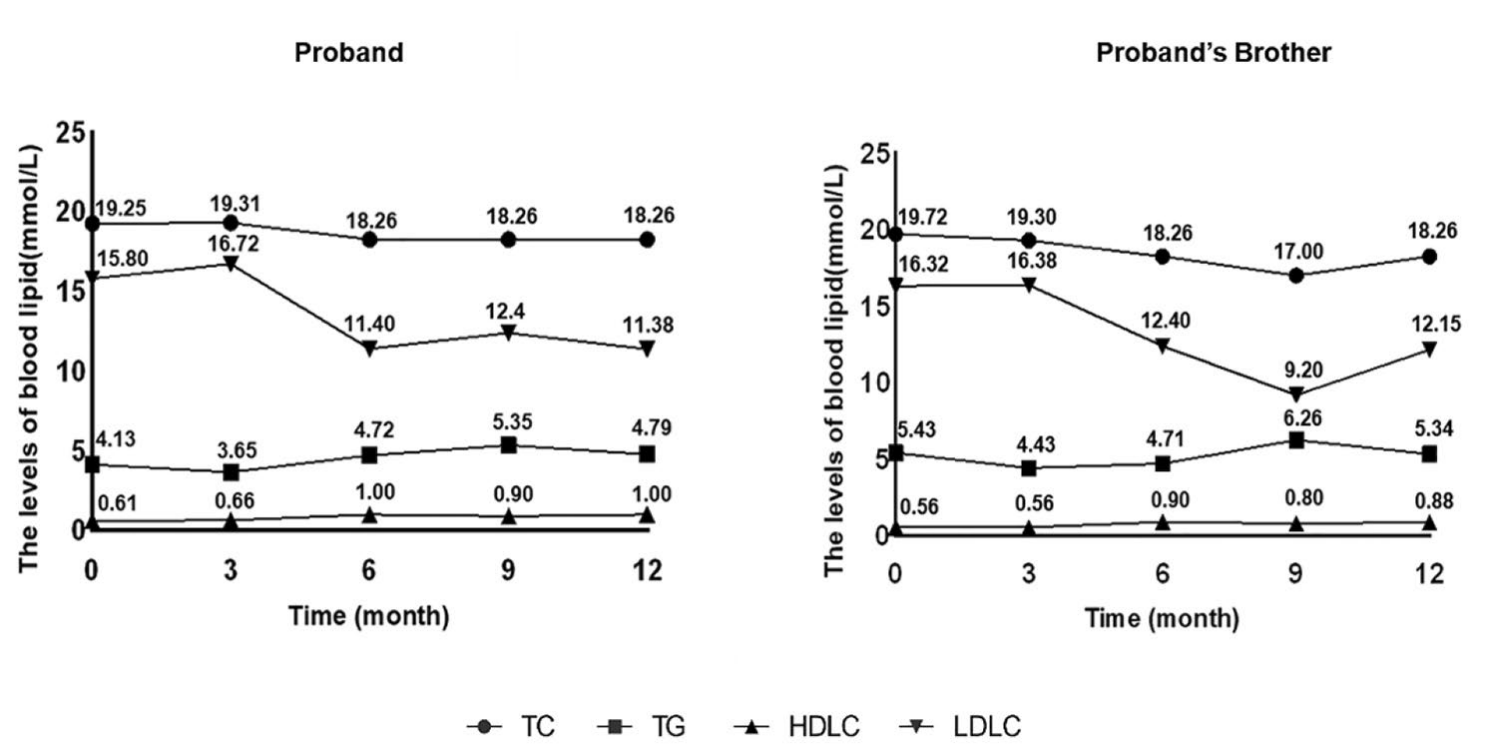
The blood lipid levels of the patients. The lipid levels of the proband and his brother were followed up for 1 year during treatment with a PCSK9 inhibitor

After 10 years of statin treatment, the proband and his brother exhibited elevated blood lipid levels. In this study, a PCSK9 inhibitor (Evolocumab, 140 mg Q2W) was used as the lipid-lowering agent. Treatment with other lipid-lowering agents remained unchanged during the Evolocumab treatment. Both patients were followed up for 1 year to evaluate their blood lipid levels, but neither the proband not his brother showed any significant benefit from PCSK9 inhibitor treatment (Fig. 5). This confirmed that PCSK9 inhibitors have limited therapeutic effects on complex gene variants associated with FH.

## Discussion

In the present study, a newly discovered frameshift insertion was identified in the *LDLR* gene. This mutation (c.497delinsGGATCCCCCAGCTGCATCCCCCAG: p. Ala166fs) results in the premature appearance of a stop codon, leading to the termination of protein translation within exon 4. Studies have reported that the *LDLR* gene accounts for 90% of FH pathogenic gene mutations, with 77% caused by base substitutions, 16% by base deletions, and 5% by duplications [16]. A meta-analysis of Chinese patients with FH indicated that *LDLR* mutations are mainly missense mutations located in exon 4 [17]. The mutated protein contained only 185 amino acids, compared to 860 amino acids in the normal LDLR protein. This mutation is considered more complex and severe than most previously reported single-gene mutations.

Protein signal peptides are typically located at the N-terminus and play an important role in directing newly synthesized proteins to their correct cellular locations. The translation of signal peptides is crucial for the localization, folding, and functional formation of newly synthesized proteins [18]. SignalIP prediction suggested that the mutated sequence contained a complete signal peptide sequence, indicating the feasibility of translation. TMHMM is a commonly used method for predicting the transmembrane structures of membrane proteins [14]. This method revealed that the normal LDLR protein contains a transmembrane domain, whereas the mutated protein lacks this domain. As LDLR is a transmembrane protein, the absence of a transmembrane region suggests that the mutant protein may be unable to traverse the cellular membrane structure to perform its biological function. SWISS-MODEL, a method for three-dimensional protein structure prediction, revealed significant structural changes in the sequence due to premature termination of translation [19]. Western blotting confirmed that the mutant fragment lacked the major structural domain of LDLR. Only correctly folded and modified mature proteins are released from the endoplasmic reticulum, which provides quality control over protein translation [20, 21]. Whether a mutated protein can be expressed by an organism depends on the specific type and location of the mutation, as well as the structural and functional changes that occur in the protein after the mutation [22]. Based on bioinformatics predictions and in vitro experiments, this mutation is believed to prevent the mutant protein from being expressed on the cell membrane. Premature termination leads to the deletion of large sequence segments, which hinders proper folding and modification.

A missense mutation c.2054C>T (p. Pro685Leu) in exon 14 of the *LDLR* gene was reported as a pathogenic mutation in a previous study [23]. Although this exon does not directly encode a specific protein functional domain, its main role is to assist adjacent exons in facilitating the proper folding of the LDLR protein and maintaining its function in the extracellular uptake of LDL particles.

In addition to the *LDLR* gene, *APOB* is considered the second most commonly implicated gene in FH [4]. Variants in exons 26 and 29 of the *APOB* gene, which play a crucial role in the involvement of apoB-100 in the clearance of LDL particles, were analyzed [7]. The proband and his mother harbored the same variant (c.13013G>A: p. Ser4338Asn) in exon 29. Homology modeling revealed changes in protein conformation at the mutation site. Additionally, the WGS results revealed that the proband and his parents shared the same variant (c.6937A>G: p. Ile2313Val) in exon 26 of the *APOB* gene. Structural predictions using SWISS-MODEL for the variations located in exon 1, exon 4, exon 14, and exon 29 of the *APOB* gene revealed that the variants in exon 1 and exon 29 alter the protein’s spatial structure. Although synonymous mutations do not directly alter amino acid sequences, their cumulative effects may affect mRNA splicing, stability, and translation speed [24, 25]. Therefore, accumulation of numerous synonymous variants (*LDLR*, *APOB* and *PCSK9*) in this family may influence protein translation, structure, and function.

The mechanism of action of PCSK9 inhibitors involves reducing the concentration of PCSK9 proteins in the bloodstream, indirectly increasing the number of LDLR molecules in the cell membrane, and enhancing blood lipid clearance [26]. In the case of the proband with variants in both *LDLR* and *APOB* genes, this led to a suboptimal response to PCSK9 inhibitors. Therefore, the proband’s insensitivity to PCSK9 inhibitors can be explained by these genetic findings. This finding serves as a reminder that genetic diagnosis can provide a theoretical framework for individualized patient treatment.

Although genetic defects contribute to the development of FH, disease severity depends on LDL-C levels. The lipid levels of the patients in this study were consistent with their genetic variants. Based on the study of this family, it was observed that the clinical severity followed the pattern: father < mother < proband. According to the sequencing results, both the father and mother were double heterozygous for mutations in *LDLR* and *APOB* genes. The mother’s *LDLR* mutation is a known pathogenic mutation, and there were more *APOB* variants at key sites (exon 26 and exon 29) than in the father’s *APOB* gene variants. The proband inherited *LDLR* and *APOB* mutations from his parents and simultaneously exhibited homozygosity for the *APOB* gene variant. Therefore, the proband exhibited severe clinical symptoms and was insensitive to PCSK9 inhibitors.

### Study strengths and limitations

This study provides groundbreaking evidence by combining bioinformatics predictions and in vitro experiments to confirm the pathogenicity of a newly discovered frameshift insertion in the *LDLR* gene. This mutation hampers the proper expression of LDLR in the cell membrane, indicating that it is severely pathogenic. Furthermore, by integrating WGS and spatial structure predictions, it was suggested that variants in the *APOB* gene was closely associated with severe clinical symptoms. Consequently, this study highlights that patients with double heterozygous mutations (*LDLR* + *APOB*) exhibit reduced sensitivity to PCSK9 inhibitor therapy. This study had some limitations. First, further investigations are needed to determine co-localization with the endoplasmic reticulum or lysosomes to fully understand the expression and translation of the new mutation in *LDLR*. Additionally, there is a lack of direct evidence linking the numerous *APOB* variants observed in this family to their pathogenicity. Further research will explore the mechanisms underlying the pathogenicity of *APOB* in subsequent studies. Furthermore, the clinical significance of these newly discovered variants requires epidemiological data, which were not available in this study. Future research should explore the role of other genes implicated in FH and include a more diverse sample of patients with FH to improve epidemiological representativeness.

## Conclusion

This study identified a novel frameshift insertion (c.497delinsGGATCCCCCAGCTGCATCCCCCAG: p. Ala166fs) and a known pathogenic mutation (c.2054C>T: p. Pro685Leu) in the *LDLR* gene. Additionally, several variants were identified in the *APOB* gene. The cumulative effect of these genetic variants likely contributes to the severe clinical symptoms observed in the proband. This study highlights the potential of genetic testing for identifying primary pathogenic genes in patients with severe symptoms and suboptimal treatment outcomes. By analyzing these pathogenic genes, it is possible to establish effective treatment approaches, thereby providing a theoretical foundation for clinical decision-making. These findings underscore the need to develop new therapeutic interventions for lowering lipid levels.

### Electronic supplementary material

Below is the link to the electronic supplementary material.

Supplementary Material 1

## Data Availability

The data that support the conclusions of this investigation are accessible upon reasonable request from the corresponding author.

## Abbreviations

ACMG: American College of Medical Genetics and Genomics
APOB: Apolipoprotein B
CDS: Coding sequence
DLCN: Dutch Lipid Clinic Network
EGF: Epidermal growth factor
FH: Familial hypercholesterolemia
HDL-C: High-density lipoprotein cholesterol
LDL: Low-density lipoprotein
LDLR: Low-density lipoprotein receptor
LDL-C: Low-density lipoprotein cholesterol
LDLRAP1: Low-density lipoprotein receptor adaptor protein 1
NC: Negative control
PCSK9: Proprotein convertase subtilisin/kexin 9
PCR: Polymerase chain reaction
RT-PCR: Reverse transcription polymerase chain reaction
TC: Total cholesterol
TG: Triglycerides
WGS: Whole-genome sequencing
WT: Wild-type
