# Supplementary figures and images for: Screening and verifying the mutations in the LDLR and APOB genes in a Chinese family with familial hypercholesterolemia
Source: Lipids Health Dis. 2023 Oct 18;22:175. doi: 10.1186/s12944-023-01935-8 (PMC10585857; doi:10.1186/s12944-023-01935-8)

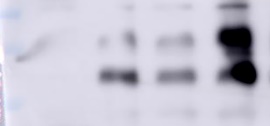

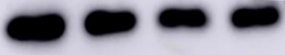

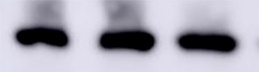

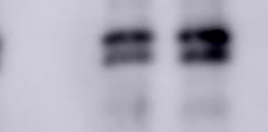
Fig 1

HEK293

HEK293

NC WT WT

NC WT WT

GAPDH

Mature LDLR

Precursor LDLR

GAPDH

Mature LDLR

Precursor LDLR

Supplement: Supplementary file 1 — Supplementary Material 1 [file 12944_2023_1935_MOESM1_ESM.docx]
